# Genome-Wide Association Study Reveals Candidate Genes Regulating Plant Height and First-Branch Height in *Brassica napus*

**DOI:** 10.3390/ijms26115090

**Published:** 2025-05-26

**Authors:** Tianyu Cui, Xinao Wang, Wenxiang Wang, Hongtao Cheng, Desheng Mei, Qiong Hu, Wenliang Wei, Jia Liu

**Affiliations:** 1 MARA Key Laboratory of Sustainable Crop Production in the Middle Reaches of the Yangtze River, Engineering Research Center of Ecology and Agricultural Use of Wetland, Ministry of Education, College of Agriculture, Yangtze University, Jingzhou 434023, China; cuitianyu523@163.com; 2 Oil Crops Research Institute, Chinese Academy of Agricultural Sciences, No. 2 Xudong 2nd Rd., Wuhan 430062, China; 3 Key Laboratory of Biology and Genetic Improvement of Oil Crops, Ministry of Agriculture and Rural Affairs, Wuhan 430062, China

**Keywords:** rapeseed, plant height, first-branch height, GWAS, candidate genes, meta-analysis

## Abstract

Rapeseed (*Brassica napus* L., 2*n* = 38) is an important oil crop worldwide, providing vegetable oil and biofuel. Despite improvements in breeding, rapeseed’s harvest index and yield remain lower than other major crops. Plant height (PH) and first-branch height (FBH) are crucial plant architecture traits affecting yield, lodging resistance and efficiency of mechanical harvesting. Phenotypic analysis of 125 rapeseed accessions across four environments revealed wide variation in PH (100–198 cm) and FBH (15.56–112.4 cm), with high broad-sense heritability (*H*^2^ = 81.59% for PH, 77.69% for FBH), and significant positive correlations between traits. To understand the genetic control of PH and FBH, a genome-wide association study (GWAS) of a natural population was conducted, covering 2,131,705 genome variants across four environments. The 13 QTLs for PH and 15 for FBH were identified. Meta-analysis revealed that 28.57% of these loci overlapped with previously reported QTLs. Haplotype analysis confirmed significant effects of these loci on the traits. Candidate genes for PH and FBH, respectively, were identified based on linkage disequilibrium and functional predictions. However, five novel loci lacked nearby annotated genes. The candidate genes are linked to traits in *Arabidopsis* and other species, as well as to phytohormone response and cell development, and cell development. Notably, *MOS1* gene copies (*BnaA03G0481200ZS* and *BnaC07G0459400ZS*) were associated with PH and FBH, indicating their multifunctional potential. Additionally, *BnaA05G0163200ZS*, with no functional annotation, emerged as a crucial gene for plant architecture. This study provides new genetic insights and may enhance marker-based breeding for ideotypes in rapeseed.

## 1. Introduction

Rapeseed (*Brassica napus* L.) is an important oilseed crop with an annual production of approximately 87 million tons (FAOSTAT, https://www.fao.org/faostat/en/, accessed on 10 June 2024), providing edible oil, crude protein feed for livestock and biofuel [1,2]. The population is predicted to surpass 9 billion by 2050, posing a challenge to rapeseed production [3]. Although breeding and cultivation measures for rapeseed have improved, the rapeseed industry still faces multiple bottlenecks, including a low yield and harvest index, low planting density and a relatively low degree of mechanization [4]. These issues seriously affect the further development of the rapeseed industry. Thus, breeding for improved plant architecture in rapeseed is highly valuable for increasing production.

Plant architecture refers to the distribution of plant organs in the field, either as individuals or populations. It includes key morphological traits such as plant height (PH), first-branch height (FBH), branch angle (BA), and other aspects that play a crucial role in determining crop yield and harvest index [5]. Both PH and FBH are essential components of rapeseed plant architecture, as they significantly impact yield and the feasibility of mechanized harvesting [6,7,8]. The positive effect of plant architecture on seed yield derives from the high plant height and low relative branching height [9]. PH plays an essential role in light interception and photosynthesis. In crop domestication, PH is the primary factor that affects crops’ lodging resistance and seed/fruit yields [10]. Although suitable plant heights in rapeseed are believed to contribute to higher yields [11,12,13], varieties with a taller stature are more prone to severe lodging, particularly under high-density planting conditions, where the problem is exacerbated [14]. Lodging damages the vascular bundles, disrupting water transport, nutrient supply, and phytohormone translocation, which in turn hinders seed formation and filling, leading to a significant reduction in both yield and quality, and remains a major challenge for rapeseed breeders worldwide [15,16]. Lower branch heights can cause winding and pulling between branches during machine harvesting, which can result in grain loss during mechanical threshing. Rapeseeds with a higher FBH have fewer branches, which helps them meet the light energy needs of rapeseed in the later stages of the season [17]. Higher branch initiation was more favorable for plant photosynthesis and angiosperm development [18]. However, a high FBH can be unfavorable for harvesting because it shifts the center of gravity upwards, making the crop more susceptible to lodging [9]. Therefore, suitable PH and FBH are key traits in rapeseed varieties for improving yield and productivity.

Modern crop breeding requirements for agronomic traits are not stationary, but depend on a variety of environments and cultivation methods [19]. Hence, understanding the genetic basis of PH and FBH will contribute to cultivating the ideal plant types to improve agronomic traits in rapeseed. Plant height (PH) and first-branch height (FBH), as quantitative traits in rapeseed, are modulated by a complex network of genetic determinants, and their intricate interactions with environmental variables [20]. This multifactorial influence underscores the complexity of breeding strategies aimed at phenotypic optimization. Previous studies have identified some loci and candidate genes for PH and FBH in rapeseed, employing quantitative trait locus (QTL) mapping to identify genetic loci associated with traits, genome-wide association studies (GWAS) for elucidating correlations between genetic polymorphisms and trait manifestations, and RNA sequencing (RNA-seq) for delineating gene expression profiles pertinent to traits [7,19,21,22]. The functions of some of the genes have been resolved. Zheng et al. (2020) identified a 20 bp deletion in the *MAX1* in a branching mutant line, which caused dwarfism and increased branching. Genetic experiments confirmed that the deletion led to a frameshift and premature stop in the *MAX1*, providing a novel allele associated with plant architecture regulation [23]. The mechanism of inheritance for some genes has been determined. Six QTLs for plant height and seven QTLs for FBH were identified using doubled haploid (DH) and intergenerational F_2_ (IF_2_) populations [20]. Fourteen QTLs for PH and five QTLs for FBH were identified in different linkage groups using a 192 rapeseed inbred lines [24]. GWAS identified *BnaA01*. *BIN2* was significantly associated with PH, and multiple allelic variations within this gene resulted in a significant reduction in PH, hinting at its pivotal regulatory capacity in the genetic trajectories of rapeseed [4]. A major QTL for PH was identified by QTL-seq and a high-density genetic map was created based on whole-genome sequencing (WGS); *BnaA10g08290D*, *BnaA10g09290D*, and *BnaA10g08230D* are potential candidate genes for this QTL [25]. Nineteen QTLs related to FBH traits distributed on five chromosomes were detected on five chromosomes using an RIL population containing 210 lines derived from the crossing of 888-5 and M083. A primary QTL for FBH on chromosome A02 was identified through the integration of linkage and association analyses. Among eleven candidate genes, *BnaA02g13010D* was confirmed as the major gene [7]. Many QTLs for PH and FBH traits in rapeseed have been identified, but the complex regulatory mechanisms have not yet been clearly investigated, and it is imperative to further explore the genetic composition of agronomic traits in rapeseed.

In this study, we measured PH and FBH in four environments using a natural population. The statistical analysis revealed that genotype and environment were the main factors influencing phenotypic variation. Both traits were significantly correlated among all environments, with a stronger correlation observed within the same environment. To explore the genes influencing PH and FBH, we conducted analysis using second-generation sequencing data in conjunction with genome-wide approaches. Significant loci and candidate genes associated with PH and FBH were identified This study helps to promote marker-based breeding of rapeseed elite lines and reveals the value of several candidate genes in the rapeseed gene pool, aiming to identify targets for breeding programs focused on optimizing rapeseed productivity and mechanization compatibility.

## 2. Results

### 2.1. Phenotypic Variations for PH and FBH

**Figure 1:**
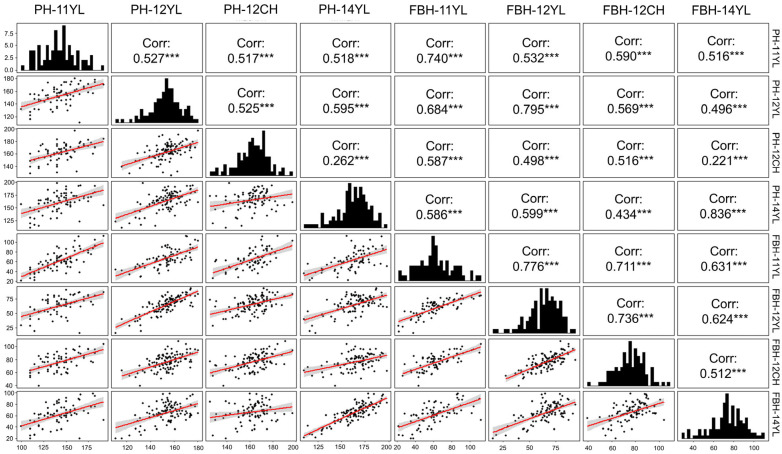
Pearson correlations and frequency distribution of PH and FBH in four environments: 11 YL, 12 CH, 12 YL, and 14 YL. The diagonal pictures show the distribution of PH and FBH. The upper right corner shows the correlation between environments. The lower left corner shows the scatter plot of PH and FBH distribution in different environments; the red line shows simple linear regression of variables. ***: *p* < 0.001 indicates extremely significant correlation.

To explore the extensive phenotypic variability of PH and FBH across environments, we quantitatively assessed two traits of 125 rapeseed accessions within four distinct environments. The phenotypic data exhibited considerable variability, with both PH and FBH distributions closely approximating a normal distribution across all environments (Figure 1). The trait of PH had 1.57–1.92-fold variations across all environment, ranging from 100 to 198 cm, and the coefficient of variation ranged from 8.26 to 13.77%. The trait of FBH had 2.75–6-fold variations across all environment, ranging from 15.56 to 112.4 cm, and the coefficient of variation ranged from 15.86 to 32.38% (Table 1). The phenotypic data for FBH had a higher level of dispersion compared to PH. In summary, the range of available variation for different traits suggested that the set of rapeseed accessions is suitable for GWAS analysis. There were significant differences in PH and FBH in rapeseed germplasm across environments, and genotypes also differed significantly for PH and FBH. However, for both traits, the genotype–environment interaction was not significant. This suggests that the expression of plant height (PH) and first-branch height (FBH) in rapeseed is primarily influenced by genetic factors, with minimal effect from environmental variations. The lack of significant genotype–environment interaction indicates that these traits exhibit stable genetic expression across different environments.

The broad-sense heritability (*H*^2^) for PH and FBH was 81.59% and 77.69% (Table 1), respectively, indicating that these two traits are stably inherited. To reveal the relationship between PH and FBH, the Pearson correlation coefficient (PCC) was used to assess the correlation between the two yield-related traits under different environments (Figure 1). PH was significantly and positively correlated with FBH in all environments. PH and FBH were significantly correlated in four environments, with correlation coefficients ranging from 0.22 to 0.84 (Figure 1). In addition, PH and FBH in the same environment showed higher correlation, with a mean PCC value of 0.72 (mean PCC value of PH and FBH in different environments was 0.53), indicating the specific regulatory effects of different environments on PH and FBH in oilseed rape.

### 2.2. Genotype Analysis

Genomic genetic variation in the natural population was detected using the filtered second-generation resequencing data, and a total of about 23.08 million variants (SNPs and InDels) were obtained. About 2,313,759 high-quality variants were obtained after deleting variants with a minimum allele frequency of less than 5%, and with a *p*-value of less than 0.01 in the Hardy–Weinberg equilibrium test, among which there were 1,873,206 SNPs and 258,499 InDels. According to reference [26], population structure refers to subpopulation differentiation within the material. Population structure analysis showed the minimum CV error at k = 1, indicating the optimal grouping was a single population. Principal component analysis also failed to divide the 125 materials into distinct groups, and both analyses concluded that no further grouping was needed.

### 2.3. Genome-Wide Association Mapping for PH and FBH

**Figure 2:**
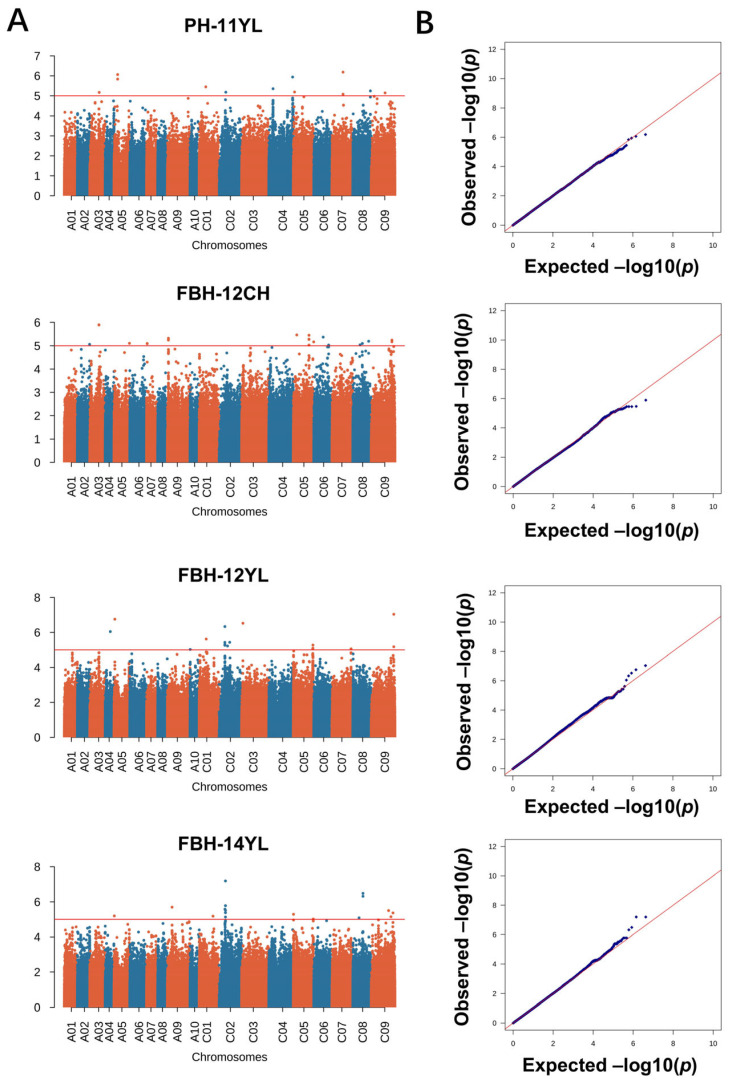
Manhattan and quantile–quantile (QQ) plots showing GWAS mapping for PH. GWAS was conducted using the mixed linear model (MLM) in Tassel v5.0. (**A**) Manhattan plot for PH across four environments (11 YL, 12 CH, 12 YL, and 14 YL); (**B**) Q-Q plot for PH across the four environments.

**Figure 3:**
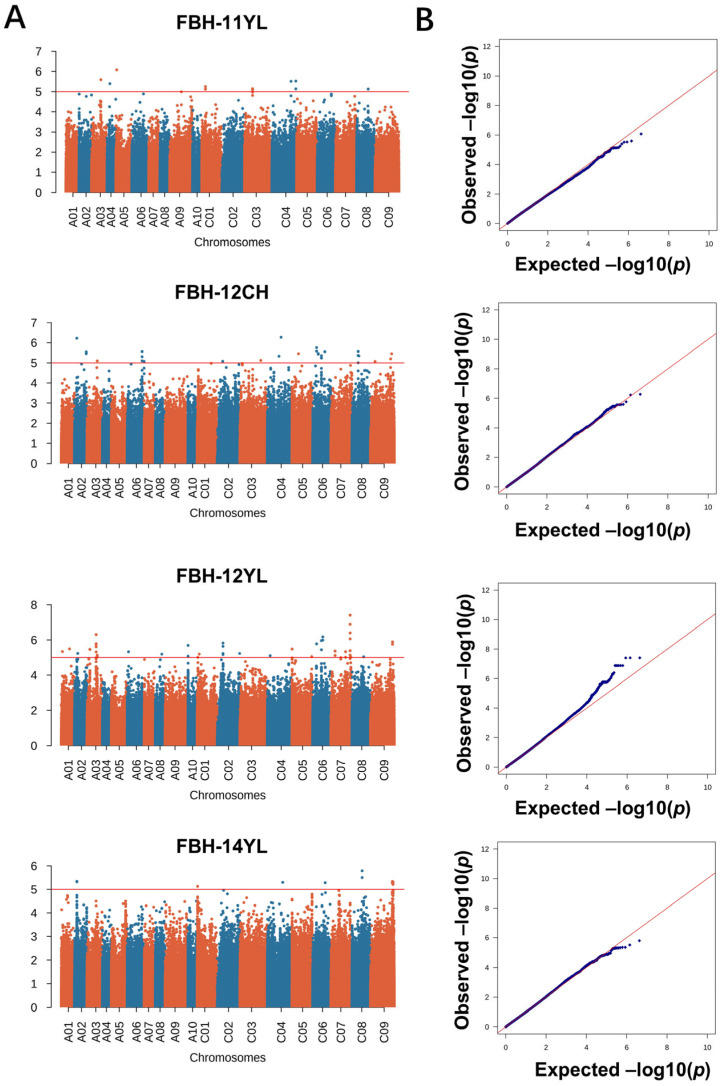
Manhattan and quantile–quantile (QQ) plots showing associations between SNPs and FBH in 125 rapeseed accessions. GWAS was conducted by the mixed linear model (MLM) in Tassel v5.0. (**A**) Manhattan plot for FBH across four environments (11 YL, 12 CH, 12 YL, and 14 YL); (**B**) Q-Q plot for the PH across four environments.

GWAS were conducted, employing 2,131,705 variants to analyze the genetic underpinnings of PH and FBH in diverse environmental settings. To dissect the complex genetic architecture of these traits, both mixed linear models (MLMs) and the Q + K model were applied, enabling an interpretation of fixed and random genetic effects. The significant deviations observed in quantile–quantile plots underscored the MLM’s efficacy in capturing the genetic variance critical for GWAS (Figure 2 and Figure 3). The rigorous association mapping process identified 156 SNPs significantly associated with the traits under study, as cataloged in Table S1. Subsequent analysis refined these findings to delineate 13 quantitative trait loci (QTL) for PH and 15 QTL for FBH, established through a significance threshold of −log_10_(*p*) > 5 (Table 2). This stringent selection criterion ensured that each QTL represented a concentration of genetic polymorphisms, thereby enhancing the precision and reliability of the association signals detected. Referring to the results of previous studies on this population, we mined candidate genes upstream and downstream of the LD decay range (r^2^ > 0.2) of significant loci.

### 2.4. Haplotype Analysis of Peak SNPs

**Figure 4:**
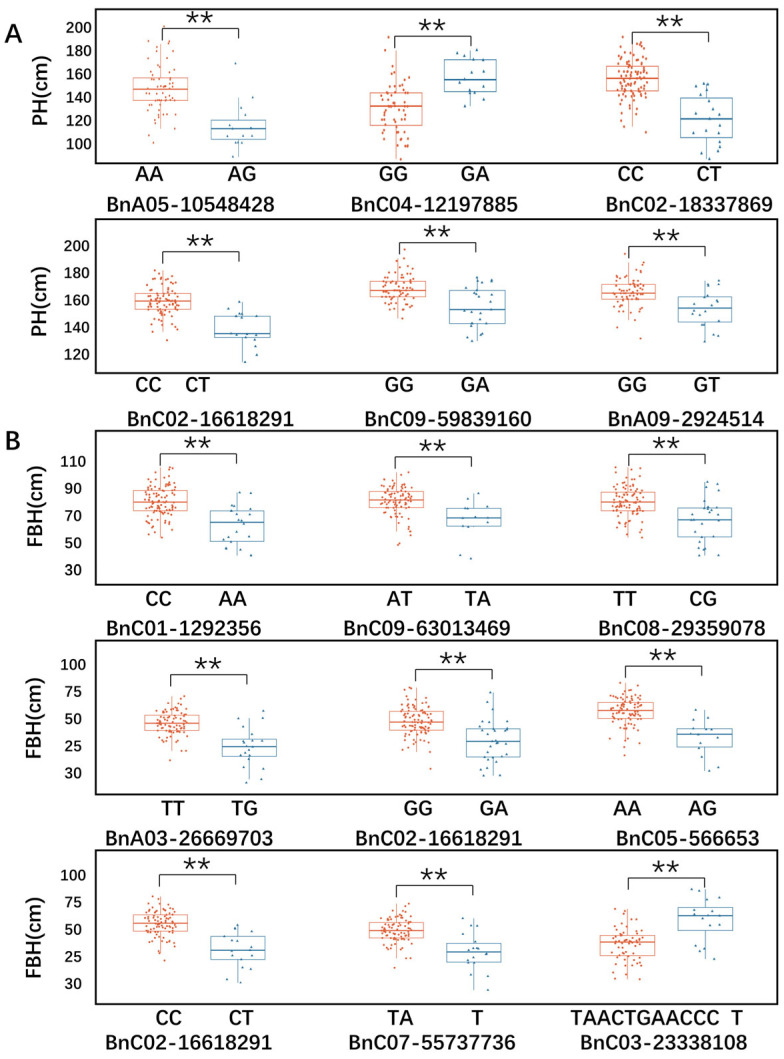
Boxplots showing the association between alleles of peak SNP markers and traits. (**A**) PH and corresponding SNP alleles; (**B**) FBH and corresponding alleles. All statistical associations are marked with and with ** (at *p* < 0.01).

Association mapping results showed the 156 significant SNPs in GWAS (Table S1). Further analysis of the 156 SNPs revealed 29 overlapping regions across different environments, as detailed in Table S2. To identify favorable alleles of QTLs for PH and FBH traits in rapeseed, we analyzed significant phenotypic differences between different alleles at the peak SNPs. The population was divided into two groups based on allele type, and the phenotypic values were compared between the groups (Figure 4). The results of the Mann–Whitney U test showed that all loci subjected to haplotype analysis were significantly affected PH or FBH (Table S3). For example, accessions with the AA SNP allele of A09:10,548,428 show higher PH compared to those with the AG variant, indicating AA can be considered as a positive effect allele for PH. Similar results were observed with other haplotype groups. These significant effects indicated the above candidate genes could be the key factors underpinning plant architecture of rapeseed. The allele frequency distribution is detailed in Table S6.

### 2.5. Gene Annotation and Candidate Gene Prediction

**Figure 5:**
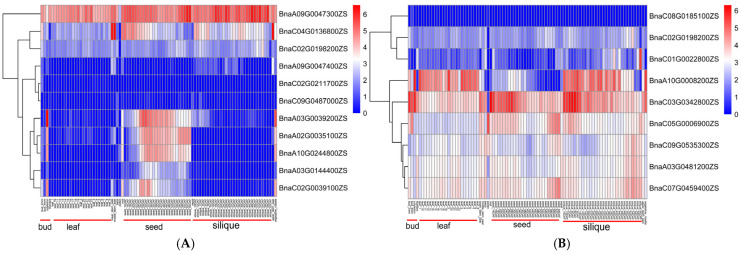
Expression pattern of candidate genes at different stages in 13 tissues of *B. napus* cv. ZS11, using log_2_ (TPM + 1) as input data for heatmap of expression of candidate genes for PH (**A**) and FBH (**B**). Tissues were sampled at developmental organs such as leaves, siliques, and silique. All data were obtained from the website (https://yanglab.hzau.edu.cn/BnIR/expression_zs11, accessed on 10 June 2024).

According to the specific physical distance o of QTLs, 30 genes for PH and 42 genes for FBH were mined from the upstream or downstream 4.5 kb region of QTL identified in different environments (Table S4). The window size of 9 kb (±4.5 kb) was chosen based on the linkage disequilibrium (LD) decay pattern observed in our natural population. The genome-wide LD decay distance was estimated at approximately 4.5 kb when the threshold of *r*^2^ = 0.2 was applied [27]. The use of a narrower window strikes a balance between capturing causal loci and avoiding inclusion of distant, potentially unrelated genes. This resolution is consistent with the LD structure of our population and improves the precision of gene annotation and candidate gene identification. Information on a total of 72 genes in the candidate region is presented (Table S4). The candidate genes were predicted by combining the reference gene annotations, the relationship of the gene to the physical location of the peak SNPs, and the results of haplotype analysis. Since PH and FBH are mainly determined by the growth and development of the main stem and meristem, we focused on genes with a higher tendency to be expressed at different locations in the stalk (Figure 5). As a result, seven and nine candidate genes associated with PH and FBH were predicted based on gene annotation and related research reports, respectively (Table 3). The expression patterns of candidate genes in tissues of Zhongshuang 11 (ZS11) were analyzed, which showed that three candidate genes for PH, including *BnaC04G0136800ZS*, *BnaA09G0047300ZS*, and *BnaA05G0163200ZS,* were expressed predominantly in the stalks (Figure 5). Except for *BnaC09G048700ZS*, *BnaC02G0211700ZS*, and *BnaC08G0185100ZS*, all the other 13 candidate genes were expressed at different stalk tissues.

### 2.6. Meta-Analysis of QTLs Controlling PH and FBH in Rapessed

**Figure 6:**
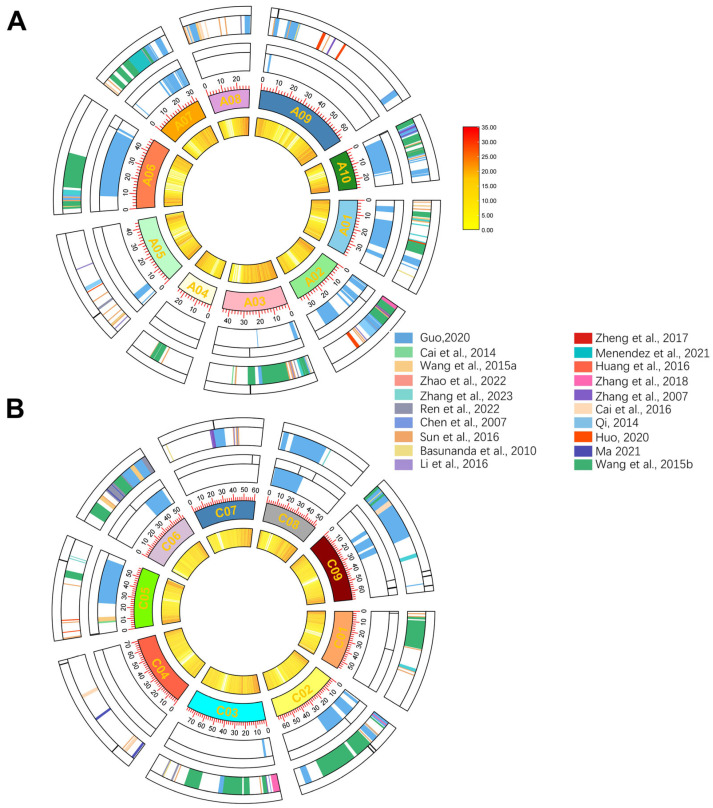
Distribution of all QTLs in the *B. napus* cultivar “ZS11” V0 reference genome. (**A**) QTLs mapped to the A sub-genome. (**B**) QTLs mapped to the C sub-genome. In both panels, the concentric loops from inside to outside indicate gene density, chromosome length (Mb), QTLs associated with FBH, and QTLs associated with PH. The colors inside the circles represent QTLs from different populations. Sources of QTLs: Chen et al., 2007 [20]; Zhang et al., 2007 [35]; Basunanda et al., 2010 [31]; Qi, 2014 [37]; Cai et al., 2014 [24]; Wang et al., 2015a [28]; Wang et al., 2015b [29]; Huang et al., 2016 [36]; Li et al., 2016 [40]; Sun et al., 2016 [30]; Cai et al., 2016 [6]; Zheng et al., 2017 [32]; Zhang et al., 2018 [34]; Guo, 2020 [27]; Huo, 2020 [38]; Menendez et al., 2021 [33]; Ma, 2021 [39]; Ren et al., 2022 [12]; Zhao et al., 2022 [4]; Zhang et al., 2023 [13].

Linkage and association analyses results obtained using Darmor or other genetic mapping genomes were converted to the relevant positions in the ZS11 genome. To gain a better understanding of the PH and FBH in rapeseed, 607 QTLs in different populations were collected from 20 studies published since 2007 (Table S5) [4,6,12,13,20,24,27,28,29,30,31,32,33,34,35,36,37,38,39,40]. The physical position of QTLs could be found in the physical map of ZS11 reference genome that finally revealed QTL regions from different populations potentially associated with PH and FBH, unequally distributed across all chromosomes (Figure 6). The comparison results showed that some of the QTL obtained in this study were located in the previous mapping interval. The mapping populations encompassed a diverse array of genetic constructs, including DH populations, recombinant inbred line (RIL) populations, nested association mapping (NAM) populations, and natural populations, as detailed in Table S5. Due to variations in chromosome sequence alignment across different genetic backgrounds, QTLs that were in close proximity were considered as overlapping. The results showed that 28.57% of the QTLs overlapped with those identified in previous studies (Table S7).

Previous studies have conducted similar QTL analyses on rapeseed traits [41,42,43], revealing comparable trends in QTL distribution. Specifically, Nadia et al. identified QTLs related to plant height (PH) predominantly on chromosomes A1, A9, C3, C6, and C9, a pattern that aligns with our own findings [42]. These studies collectively provide strong evidence for the consistency of QTL distribution across different rapeseed traits, supporting the reliability of our analysis of PH and first-branch height (FBH).

## 3. Discussion

Over the past three decades, growing interest has led to an 82% increase in oilseed acreage and a 240% increase in total world production [40]. Due to its widespread cultivation for oil production, rapeseed is also the most popular source of plant protein [2]. PH and FBH are important agronomic and architecture traits of rapeseed. Adequate plant height and lower first-branch height are important goals in rapeseed breeding [20]. To improve yield, facilitate mechanical harvesting, enhance lodging resistance, and optimize plant architecture, a more detailed analysis of the genetic basis underlying these traits is essential.

### 3.1. Novel QTLs Associated with PH and FBH

A number of QTLs were obtained through GWAS of PH and FBH in natural populations of rapeseed, from which we identified some novel SNPs and explored them in combination with meta-analyses, expression analyses, haplotype analyses, and advances of previous studies. These QTLs were distributed across both A and C subgenomes, with a higher frequency observed on chromosomes C02, C09, and A03. Notably, chromosome C02 harbored multiple QTLs for both traits, including *qBnPH.C02.1*, *qBnPH.C02.2*, *qBnPH.C02.3*, and *qBnFBH.C02*, suggesting a potential QTL cluster or hotspot region. Similarly, loci on chromosome A03 for PH (*qBnPH.A03*) and FBH (*qBnFBH.A03*) were located in close physical proximity (within ~2 kb), implying the existence of a potentially pleiotropic or co-localized QTL influencing both traits. Among the identified loci, *qBnFBH.C07* and *qBnPH.C02.3* exhibited the highest levels of significance, with –log_10_(*p*) values exceeding 7.0, indicating strong genetic associations. The repeated detection of QTLs on C09 and A03 across multiple environments further highlights these regions as genetically stable and important for the regulation of PH and FBH in *Brassica napus*. The peak SNP BnA05-10548415 was located within the first exon of *BnaA05G0163200ZS* (A05:10,547,705–10,548,463 bp), which is the only gene found in the QTL region. Haplotype analysis indicated that this locus is significantly associated with plant height. Notably, meta-analysis revealed no other relevant QTL localization results in the upstream or downstream regions of BnA05-10548415. Moreover, neither this gene nor its homologues in *Arabidopsis* have been previously studied, suggesting that *BnaA05G0163200ZS* may be a novel gene influencing plant height. We analyzed the protein sequence of 346 amino acids using the SMART online tool (http://smart.embl.de/, accessed on 15 May 2025). The analysis identified a Low Complexity Region (LCR) located between amino acid positions 34 and 49. No other conventional domains, repeats, or motifs were predicted within the sequence (Figure S2). Through protein BLAST comparison, we found that many sequences align to Brassica species, such as *Brassica napus*, *Brassica oleracea*, *Brassica rapa*, and *Brassica cretica*. Other species include *Arabidopsis thaliana*, *Camelina sativa*, *Sinapis alba*, and *Raphanus sativus*, among others. These alignments suggest that the proteins are highly conserved across different plant species, with potential similarities in function and structure (Figure S3).

In qBnPH.C09.2, we identified *BnaC09G0487000ZS*, a gene encoding a protein of unknown function related to cortical microtubule organization, which has not been correlated with studies on plant height. Nevertheless, in *Arabidopsis*, a mutant of the homologous gene *AT3G55005* exhibits an abnormal cell growth and division pattern [44]. Protein domain analysis revealed no significant domains, repeats, or conserved motifs. However, it identified an IENR1 domain (positions 3–60) with a high E-value (1466.99), suggesting a possible DNA-binding role via a helix-turn-helix motif. This domain is a repeat of unknown function, potentially involved in DNA interaction (Figure S2).

Additionally, in the analysis of the loci, five loci lacked subsequent information—either no genes were found upstream or downstream, or only a single gene was present without a homolog or functional annotation in *Arabidopsis thaliana*, which suggests a potential gap in the current genomic data or a need for further exploration to fully characterize these regions.

### 3.2. Further Functional Analyses of Candidate Genes

In addition to the genes already mentioned above, there are a number of candidate genes with classical or to be further discussed functions. Different copies of *MOS1* were screened by GWAS in different environments and for different traits. *BnaA03G0481200ZS* and *BnaC07G0459400ZS* are homologous to *MOS1*, which encode Protein MODIFIER OF SNC1 1. *MOS1* performs various regulatory activities in multiple aspects of plant growth, including the flowering time, cell cycle, and stress responses in *Arabidopsis*. Since *MOS1* functions closely with *TCP15*, *TCP1* has the same role as *TCP15*; we hypothesize that *BnaA03G0481200ZS* and *BnaC07G0459400ZS* (*MOS1*) are also associated with BR synthesis [45,46].

Phytohormones such as auxin, brassinosteroids (BRs), and gibberellins (GAs) have significant effects on plant architecture. In the interval of qBnPH.C04, *BnaC04G0136800ZS* encodes transcription factor *IAA13*, which is highly expressed in the stem. Overexpression of *IAA13* in Arabidopsis and Oryza sativa resulted in slower plant growth and less lateral root formation [47,48]. Overexpression of *IAA13* in Eucalyptus and Populus positively regulates the development of xylem fibers and ducts [49]. Notably, *BnIAA7* mutations affect plant morphology, yielding compact, high-yield dwarf mutants [50]. *BnaC04G0136800ZS* (*IAA13*), mainly expressed in stems, alters growth and root formation in various plants [47,48,49,50]. Similarly, in rapeseed, *IAA13* may regulate the growth hormone-responsive transcriptome through the IAA13-ARF module, thereby affecting plant stem development. GAs are a class of phytohormones belonging to the group of diterpenoids, which play an important role throughout the life cycle of plants, promoting cell division and elongation, hypocotyl and stem elongation, and root growth [51]. The candidate gene *BnaA09G0047400ZS* (*SLY2*) for PH is the key component of the GA signaling pathway which is involved in the degradation of the Della protein by forming complexes with other proteins [52]. The candidate gene *BnaC01G0022800ZS* for FBH is regulated by the jasmonate pathway, which can strongly induce TIFY family genes, which may negatively regulate branching development [53]. *BnaC02G0211700ZS* (*TCP1*) activates downstream BR synthesis genes [45].

Among the identified QTLs, most could be associated with specific candidate genes based on their physical positions. However, one notable exception was *qBnPH.A09*, where the peak SNP (2,924,514 bp) was located between two genes—*BnaA09G0047400ZS* and *BnaA09G0047300ZS*—making it difficult to determine a single definitive candidate. Both genes are plausible regulators of plant height. *BnaA09G0047400ZS* encodes an F-box protein with a protein sequence similar to *SLY1*, a member of the SCF-SLY1 E3 ligase complex. The SCF-SLY1 E3 ligase is responsible for degrading DELLA proteins, which are involved in promoting growth. Overexpression of *SLY2* can partially compensate for the dwarfism phenotype observed in the sly1-10 mutant [47,54]. Additionally, *BnaA09G0047300ZS* encodes a nuclear PHD finger protein that is functionally redundant with *OBE1* and plays a critical role in maintaining and/or establishing root and shoot apical meristems [48]. This peak SNP may regulate rapeseed plant height by affecting the expression of either gene, or potentially through a synergistic effect between the two genes.

### 3.3. Visualizing QTL Through Meta-Analysis

Through in-depth QTL analysis and meta-analysis, more valuable information was obtained. In this study, overlapping loci were identified across different environments. Specifically, the QTLs qBnPH.A03 and qBnFBH.A03, located in the 12 CH and 12 YL environments, respectively, share SNPs that are only 1.6 kb apart and both lie within the gene sequence of the candidate gene *BnaA03G0481200ZS*. Interestingly, although the same loci for the same trait were not identified across multiple environments, homologous candidate genes were inferred. The QTL qBnFBH.C07, derived from the BnaC07-55737736 locus, along with qBnPH.A03 and qBnFBH.A03, all point to different copies of the MOS1 gene. The peak SNP for this QTL is also located within the gene sequence.

Meta-analysis of QTLs integrates results from multiple studies to identify consistent loci associated with specific traits across populations [55]. This approach, widely applied in crops like rice, enhances statistical power and supports breeding by detecting robust QTLs [56]. In oilseed rape, numerous studies on plant height and first-branch height over the past two decades used linkage and association analyses. We selected studies from 2007 onward, considering marker types and map density, and visualized valid QTLs using Circos plots. Among the 13 plant height QTLs and 15 branch height QTLs identified in this study, 7 plant height QTLs did not overlap with previously reported loci, representing novel QTL loci for oilseed rape plant height. Additionally, most of the branching height QTL loci did not coincide with findings from earlier studies, which may be partly attributed to the relatively limited number of localization studies conducted for this trait.

### 3.4. QTL Detection: Effects of Background and Sample Size

Genetic background differences, including mutations, gene order, and genotype–environment interactions, can affect QTL overlap rates. Mutations and chromosomal rearrangements can shift allele frequencies and QTL positions, resulting in inconsistent overlap rates, especially when using different reference genomes. These genomic differences contribute to mapping inconsistencies across studies, further complicating QTL identification and comparison. To more accurately detect variants and reduce potential phenotypic errors, we used an association panel of 125 rapeseed accessions. Although the sample size is relatively small, this panel exhibits high phenotypic diversity and suitable genetic background, and has been successfully used in mapping several important genes. While a smaller population may reduce QTL detection power, it also minimizes phenotyping errors, ensuring the reliability of our results.

## 4. Materials and Methods

### 4.1. Plant Materials

The association population used in this study consisted of 125 diverse rapeseed accessions [57], which were systematically cultivated in four distinct environmental settings to assess the extent of genetic diversity and the interaction between genotype and environment on phenotypic variations. The environments selected for cultivation included Yangluo City, Hubei Province (30° N, 114° E), during three separate years—2011 (11 YL), 2012 (12 YL), and 2014 (14 YL)—and Chaohu City, Anhui Province (32° N, 118° E), in the year 2012 (12 CH). These sites, located in the southern Jianghuai Hills and eastern Jianghan Plain, respectively, represent typical environmental conditions for rapeseed cultivation, providing a basis for this analysis (Figure S1). To mitigate the influence of environmental heterogeneity on trait expression, the experimental design was articulated as a completely randomized block system, implemented with dual replicates across each environmental condition. Each line of the population was allocated to a designated plot, structured into three rows, each containing 54 plants. A spatial arrangement was maintained with an inter-row spacing of 33 cm and an intra-row plant spacing of 11 cm, culminating in a planting density of 270,000 plants per hectare. Comprehensive management of the experimental plots adhered to prevailing local field management standards and cultivation practices, ensuring that the observed phenotypic variability could be attributed with confidence to genetic differentiation among the accessions rather than to variances in agronomic treatment.

### 4.2. Phenotyping and Data Analysis

At maturity, the association population was evaluated for PH and FBH in each environment. Ten representative plants in the middle of each plot were selected. PH refers the height from the base of the stem to the tip of the main inflorescence. FBH was measured as the height from the base of the stem to the first effective branch which defined as a branch originating from an axillary bud that develops into a seed-bearing branch. Phenotypic variation in the four environments was analyzed using SPSS 26.0. General linear models were constructed using genotypic, environmental, and phenotypic data, to calculate genotypic effect significance (*G*), environmental effect significance (*E*), and genotype × environment interaction effect significance (*G* × *E*). The broad-sense heritability (*H*^2^) of PH and FBH was calculated as follows:

$$H^{2}=G/(G+\frac{G\times E}{n}\;\frac{e}{nr})$$

where *G* is the genetic variance, *G* × *E* is the variance of the genotype–environment interaction, *e* is the residual error variance, *n* is the number of environments, and *r* is the number of replicates within the environment [58].

### 4.3. Genotyping Data Processing

Files of the resequencing data for the natural population were downloaded from the GSA website (https://ngdc.cncb.ac.cn/gsa/, accessed on 7 April 2024) under project number CRA013310 [26]. The population was sequenced to a depth of 10×, and it is based on Illumina HiSeq resequencing data. The population was sequenced on the Illumina HiSeq platform with an average depth of 10×. After variant calling and quality control, a total of 2,131,784 high-quality SNPs and InDels were obtained for subsequent GWAS analysis [59,60].

### 4.4. Genome-Wide Association Analyses

Population structure was calculated using admixture software (Version 1.3.0), taking k = 1~5 to view CV (Cross-Validation) error directly using shell command line. The population structure file that exhibited the lowest CV error was chosen as the input for the GWAS analysis. Population structure was used as a cofactor in GWAS, using the findings from the structural analyses reported in Yang et al. [53]. PopLDdecay software v3.43 [10] was used to estimate the LD (linkage disequilibrium) decay distance on the chromosomes of the natural population.

Associations between phenotypes and genotypes in the four environments were detected using multi-environment mixed linear models (MLMs) in the Tassel 5.0 (http://www.maizegenetics.net/tassel, accessed on 15 April 2024). The kinship matrix was added to the model to eliminate false positives caused by kinship, and finally EMMAX software (emmax-beta-07Mar2010) was used for MLM analysis. Manhattan plots and quantile–quantile plots (QQ plots) were mapped in R4.3.0 [61] using the qqman package v0.1.8 (https://cran.r-project.org/web/packages/qqman/index.html, accessed on 15 May 2025) [62]. In this study, the *p*-value indicates whether the SNP is associated with the corresponding trait or not. The criterion for significant loci obtained by GWAS is *p* < 1 × 10^−5^. The physical location of each QTL was determined based on significant loci determined by *p*-values and LD decay distances calculated from resequencing data.

### 4.5. Gene Functional Annotation

Considering the LD decay distance of the rapeseed population, the regions within 4.5 kb on either side of the QTLs based on GWAS were used to identify the candidate genes [33]. To further determine the candidate genes associated with PH and FBH, we analyzed transcriptomic data of candidate genes from Expression profile (ZS11 library) [26]. Candidate genes were annotated in the NR (https://fip.ncbi.nlm.nih.gov/blast/db/FASTA, accessed on 24 April 2024) and Swiss-Port (https://www.expasy.org/resources/uniprotkb-swiss-prot, accessed on 25 April 2024) database using blast. The function of homologous genes in *Arabidopsis thaliana* was screened and checked on the TAIR website (https://www.arabidopsis.org/index.jsp, accessed on 28 April 2024).

### 4.6. Assessing Allelic Contributions to PH and FBH

To elucidate the impact of haplotypes within candidate genes on PH and FBH, this study identified variant sites associated with peak loci and candidate genes from the GWAS findings for comprehensive haplotype analysis. The allele exerting a more pronounced influence on the phenotypic trait was designated as the dominant allele. Given the considerable size of the sample pool, the Mann–Whitney U test, a nonparametric statistical method, was employed to discern significant phenotypic variations between haplotype groups. For visual representation of the phenotypic distribution across haplotypes, box plots were generated utilizing the ggplot2 package [63] in the R4.3.0.

### 4.7. Integration of Localization Insights for PH and FBH in Rapeseed

In this study, results from linkage and genome-wide association studies on PH and FBH traits in rapeseed were systematically compiled and integrated. This relied on data from 20 studies published over the past 15 years [6,7,12,13,20,24,35,36,37,38,39,40,41,42,43,44,45,46,47]. For the linkage analysis outcomes, we determined the physical positions of QTL regions by aligning the sequences of flanking primers with the *B. napus* genome [64]. The localization involved utilizing blast searches (http://yanglab.hzau.edu.cn/BnIR/BLAST, accessed on 28 April 2024) and electronic PCR (E-PCR) (https://yanglab.hzau.edu.cn/BnIR/ePCR, accessed on 29 April 2024) to map the markers described in these studies onto the ZS11 reference genome. Since some markers could not be located on the map, we took a region of 1 Mb to delimit these QTLs from one marker. In addition, GWAS findings were contextualized within ±100 kb intervals surrounding the significantly associated SNPs. For loci used in the *Darmor* reference genome unlocated primer sequences, their physical location in the ZS11 reference genome was determined by BLAST by extracting sequences near the physical location. Ultimately, these comprehensive localization efforts culminated in the alignment of all results with the ZS11 reference genome (zs11.v0), facilitating a unified representation of the genetic underpinnings of PH and FBH in rapeseed.

## 5. Conclusions

This study offers significant insights into the genetic architecture of PH and FBH in rapeseed, which are essential traits influencing yield, mechanical harvesting, and plant stability. Through a comprehensive GWAS spanning four environments, 13 QTLs for PH and 15 for FBH were identified, with several novel loci not previously reported. These loci were validated through meta-analysis, revealing their stability and potential utility in breeding programs aimed at improving rapeseed architecture. Notably, candidate genes linked to PH and FBH include copies of the *MOS1* (*BnaA03G0481200ZS* and *BnaC07G0459400ZS*), which play multifunctional roles in plant development, and the unannotated gene *BnaA05G0163200ZS*, which emerged as a key factor in regulating plant architecture. The identification of these loci and genes provides a valuable genetic foundation for marker-assisted selection in rapeseed breeding. Furthermore, the study underscores the importance of integrating multiple analytical approaches, such as meta-analysis and haplotype analysis, to uncover robust and novel genetic loci with practical breeding applications. These findings contribute to advancing our understanding of the genetic regulation of PH and FBH in rapeseed, offering a promising direction for the development of high-yield, mechanically harvestable, and disease-resistant rapeseed varieties.

## Figures and Tables

**Table 1:** Summary statistics of the phenotypic variation in plant height (PH) and first-branch height (FBH) in the 125 rapeseed accessions grown across four environments (YL: Yangluo; CH: Chaohu).

Traits	Envirment	Min (cm)	Max (cm)	Mean b (cm)	SD	Var	CV (%)	G	E	G × E	*H*^2^%
PH	2011 YL	100.00	192.00	143.39	19.74	394.93	13.77	0.000 **	0.000 **	0.145	81.59%
	2012 YL	110.53	180.23	153.11	13.05	170.23	8.52				
	2012 CH	125.30	197.00	163.09	13.47	181.47	8.26				
	2014 YL	110.70	198.00	163.17	17.49	308.36	10.72				
FBH	2011 YL	21.60	112.40	62.27	20.16	406.55	32.38	0.000 **	0.000 **	0.835	77.69%
	2012 YL	15.56	93.49	64.62	14.83	220.03	22.95				
	2012 CH	39.50	108.80	76.83	12.19	148.51	15.86				
	2014 YL	29.70	108.60	74.12	16.35	267.45	22.06				

CV (coefficient of variation), G: genotype effect, E: environmental effect, G × E: genotype and environment interaction effect. ** (*p* < 0.01). *H*^2^ is the heritability of the population.

**Table 2:** QTL related to PH and FBH were identified by whole-gene association analysis in 125 *Brassica napus* materials. Physical location refers to ZS11 reference genome. The standard of QTL identification is −log_10_(*p*) > 5.

Traits	QTL	Environment	SNP Information	Chr	−log_10_(*p*) Value	Physical Position (bp)
PH	qBnPH.A05	11 YL	BnA05-10548415	A05	6.06292651	10,548,428
	qBnPH.C04.1	11 YL	BnC04-12197885	C04	5.35438533	12,197,885
	qBnPH.C04.2	11 YL	BnC04-69054635	C04	5.93871751	69,054,635
	qBnPH.C07	11 YL	BnC07-32385832	C07	6.18474012	32,385,849
	qBnPH.A03	12 CH	BnA03-26668095	A03	5.8933031	26,668,095
	qBnPH.A09	12 CH	BnA09-2924514	A09	5.32251692	2,924,514
	qBnPH.C05	12 CH	BnC05-45487644	C05	5.45243419	45,487,682
	qBnPH.C09.2	12 CH	BnC09-59839160	C09	5.24223678	59,839,160
	qBnPH.C02.1	12 YL	BnC02-16589091	C02	6.33308716	16,618,291
	qBnPH.C09.3	12 YL	BnC09-65242550	C09	7.0368198	65,242,550
	qBnPH.C02.2	14 YL	BnC02-18325320	C02	5.51436998	18,325,630
	qBnPH.C02.3	14 YL	BnC02-18337801	C02	7.19441013	18,337,869
	qBnPH.C09.1	14 YL	BnC09-56950142	C09	5.16269108	56,950,142
FBH	qBnFBH.C03	11 YL	BnA03-27045466	A03	5.59538325	27,045,466
	qBnFBH.A06	12 CH	BnA06-42775073	A06	5.56208905	42,775,086
	qBnFBH.C06	12 CH	BnC06-25425361	C06	5.36219091	25,425,373
	qBnFBH.C08.1	12 CH	BnC08-17906537	C08	5.56745805	17,906,542
	qBnFBH.C09.1	12 CH	BnC09-57958042	C09	5.20144207	57,958,080
	qBnFBH.A03	12 YL	BnA03-26665934	A03	6.30674196	26,665,934
	qBnFBH.A10	12 YL	BnA10-417971	A10	5.69221294	417,971
	qBnFBH.C02	12 YL	BnC02-16617997	C02	5.82304965	16,617,997
	qBnFBH.C05	12 YL	BnC05-566653	C05	5.48276675	566,653
	qBnFBH.C07	12 YL	BnC07-55737453	C07	7.40440208	55,737,453
	qBnFBH.C09.3	12 YL	BnC09-63425475	C09	5.88970586	63,425,475
	qBnFBH.A02	14 YL	BnA02-7019008	A02	5.34107964	7,019,015
	qBnFBH.C01	14 YL	BnC01-1292356	C01	5.12742125	1,292,356
	qBnFBH.C08.2	14 YL	BnC08-29359074	C08	5.78943439	29,359,078
	qBnFBH.C09.2	14 YL	BnC09-63013243	C09	5.33866552	63,013,469

**Table 3:** Detailed information of QTL. QTLs related to PH and FBH were identified by whole-gene association analysis in 125 *Brassica napus* materials. Under the threshold of R^2^ = 0.2, the attenuation distance of the whole genome is 4.5 Kb, and the QTL region range is increased by 4.5 Kb in the upstream and downstream of the original region.

Traits	QTL	Candidate Gene ID	Gene Position (bp)	Ath Homolog	Gene Symbol	Gene Annotation
PH	qBnPH.A05	*BnaA05G0163200ZS*	A05:10,547,350–10,548,463	*AT1G53180*	NA ^a^	Unannotated
	qBnPH.C02.3	*BnaC02G0211700ZS*	C02:18,337,482–18,338,522	*AT1G67260*	*TCP1*	Transcription factor TCP1
	qBnPH.C04.1	*BnaC04G0136800ZS*	C04:12,190,541–12,191,612	*AT2G33310*	*IAA13*	Auxin-responsive protein IAA13
	qBnPH.A09	*BnaA09G0047400ZS*	A09:2,921,978–2,922,436	*AT5G48170*	*SNE*	Encodes an F-box protein whose protein sequence is similar to SLY1
		*BnaA09G0047300ZS*	A09:2,917,591–2,920,949	*AT5G48160*	*OBE2*	Encodes a nuclear PHD finger protein
	qBnPH.C09.2	*BnaC09G0487000ZS*	C09:59,837,097–59,837,300	*AT3G55005*	*TON1B*	Encodes protein TONNEAU 1b, which is involved in cortical microtubule organization
	qBnPH.C02.1	*BnaC02G0198200ZS*	C02:16,615,930–16,619,295	*AT1G65080*	*ALB3L2*	Membrane insertion protein ALBINO3-like protein 2
FBH	qBnFBH.C09.2	*BnaC09G0535300ZS*	C09:63,011,337–63,013,676	*AT5G12230*	*MED19A*	Mediator of RNA polymerase II transcription subunit 19a
	qBnFBH.C08.2	*BnaC08G0185100ZS*	C08:29,352,671–29,355,012	*AT1G54560*	*XI-E*	MYO11C2, encodes a class XI myosin
	qBnFBH.C03	*BnaC03G0342800ZS*	C03:23,336,848–23,337,775	*AT5G15320*	NA	Unannotated
	qBnFBH.A03	*BnaA03G0481200ZS*	A03:26,664,082–26,671,174	*AT4G24680*	*MOS1*	Encodes Protein MODIFIER OF SNC1 1
	qBnFBH.A10	*BnaA10G0008200ZS*	A10:412,537–413,602	*AT5G19550*	NA	RNA-binding (RRM/RBD/RNP motifs) family protein
	qBnFBH.C02	*BnaC02G0198200ZS*	C02:16,615,930–16,619,295	*AT4G24680*	*MOS1*	Homologous to ALB3L2
	qBnFBH.C07	*BnaC07G0459400ZS*	C07:55,733,820–55,740,425	*AT1G01080*	NA	Encodes Protein MODIFIER OF SNC1 1
	qBnFBH.C01	*BnaC01G0022800ZS*	C01:1,289,753–1,290,852	*AT1G65080*	*ALB3L2*	Calcium uniporter protein 1
	qBnFBH.C05	*BnaC05G0006900ZS*	C05:566,307–570,741	*AT1G01510*	*AN*	Encodes a C-terminal binding protein AN

^a^ NA indicates unannotated.

## Data Availability

All data generated or analyzed during this study are included in this published article and its Supplementary Materials.

## Supplementary Materials

The following supporting information can be downloaded at https://www.mdpi.com/article/10.3390/ijms26115090/s1.
