# Supplementary material for: Genome-Wide Association Study Reveals Candidate Genes Regulating Plant Height and First-Branch Height in Brassica napus
Source: Int J Mol Sci. 2025 May 26;26(11):5090. doi: 10.3390/ijms26115090 (PMC12155442; doi:10.3390/ijms26115090)
Supplement: Supplementary file 1 [file ijms-26-05090-s001.zip › Figures S1-S3.pdf]

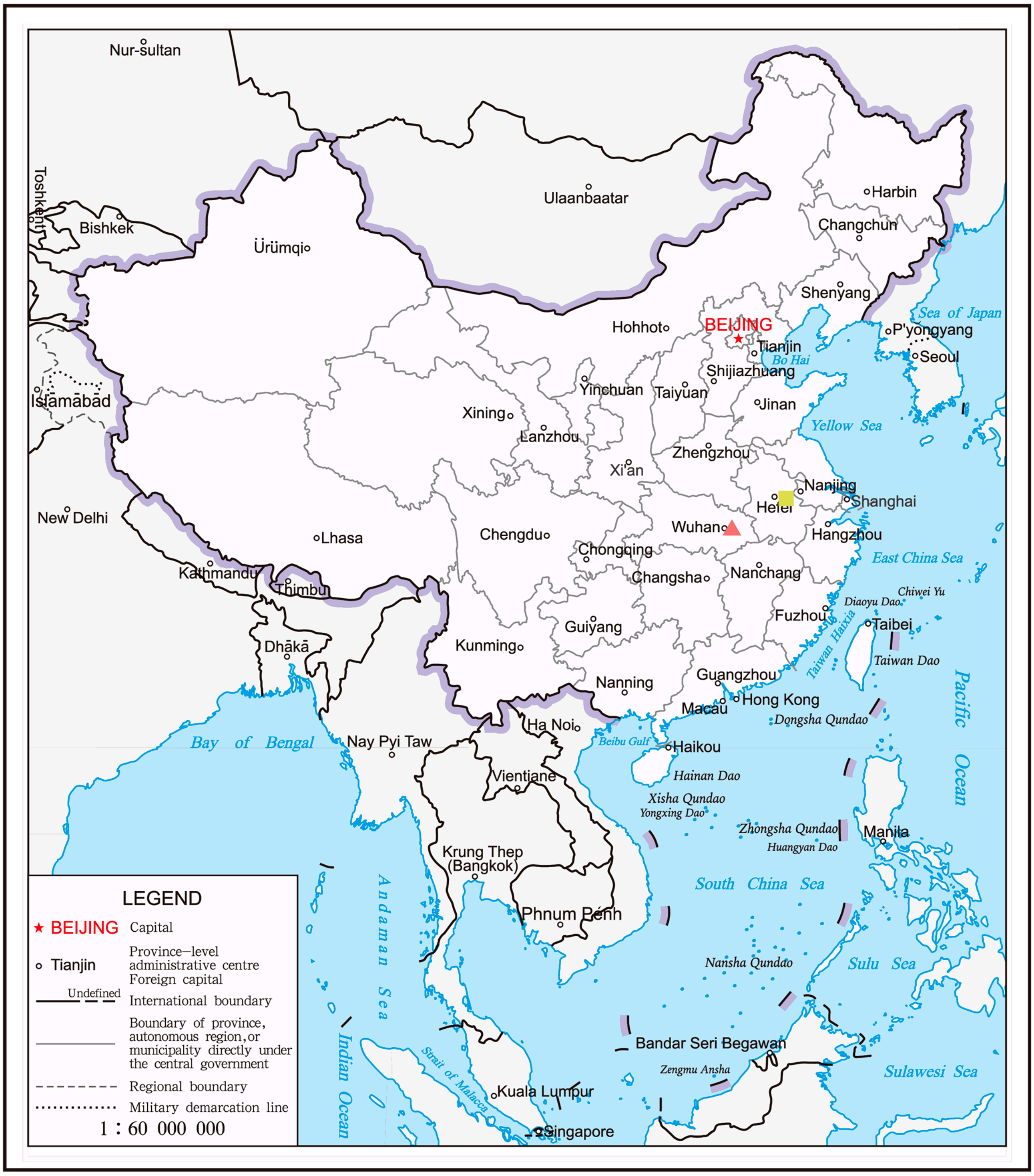

Figure S1. Geographical locations of the natural population in two distinct environments: Yangluo City (Hubei) and Chaohu City (Anhui), Yangluo city marked with a red triangle and Chaohu city marked with a yellow square.

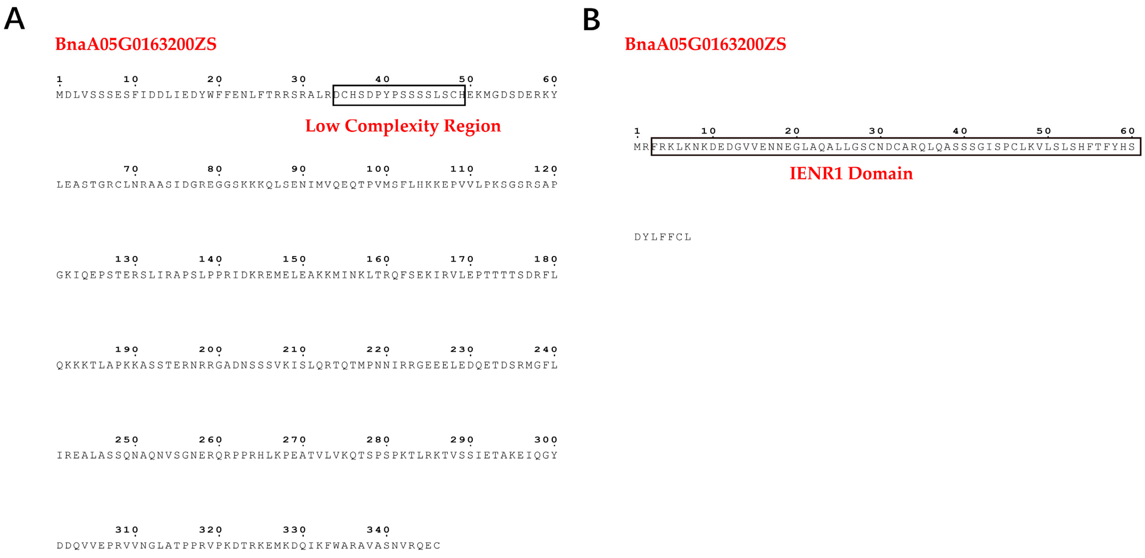

Figure S2. Protein Domain Analysis.

Organism	Blast Name	Score	Number of Hits	Description
Brassicales	eudicots		152	
. Brassicaceae	eudicots		151	
. . Brassiceae	eudicots		92	
. . . Brassica	eudicots		67	
. . . . Brassica napus	eudicots	710	25	Brassica napus hits
. . . . Brassica rapa	eudicots	710	8	Brassica rapa hits
. . . . Brassica oleracea var. oleracea	eudicots	651	3	Brassica oleracea var. oleracea hits
. . . . Brassica carinata	eudicots	651	19	Brassica carinata hits
. . . . Brassica oleracea	eudicots	651	3	Brassica oleracea hits
. . . . Brassica cretica	eudicots	649	7	Brassica cretica hits
. . . . Brassica rapa subsp. trilocularis	eudicots	442	2	Brassica rapa subsp. trilocularis hits
. . . Hirschfeldia incana	eudicots	607	5	Hirschfeldia incana hits
. . . Raphanus sativus	eudicots	599	10	Raphanus sativus hits
. . . Sinapis alba	eudicots	541	4	Sinapis alba hits
. . . Eruca vesicaria subsp. sativa	eudicots	522	6	Eruca vesicaria subsp. sativa hits
. . Thlaspi arvense	eudicots	503	2	Thlaspi arvense hits
. . Arabidopsis arenosa	eudicots	441	1	Arabidopsis arenosa hits
. . Arabidopsis thaliana	eudicots	438	20	Arabidopsis thaliana hits
. . Eutrema salsugineum	eudicots	435	4	Eutrema salsugineum hits
. . Cardamine amara subsp. amara	eudicots	435	3	Cardamine amara subsp. amara hits
. . Arabidopsis thaliana x Arabidopsis arenosa	eudicots	436	3	Arabidopsis thaliana x Arabidopsis are
. . Microthlaspi erraticum	eudicots	429	2	Microthlaspi erraticum hits
. . Arabidopsis lyrata subsp. lyrata	eudicots	427	4	Arabidopsis lyrata subsp. lyrata hits
. . Arabidopsis lyrata	eudicots	427	2	Arabidopsis lyrata hits
. . Arabis alpina	eudicots	427	2	Arabis alpina hits
. . Camelina sativa	eudicots	415	6	Camelina sativa hits
. . Arabidopsis suecica	eudicots	411	3	Arabidopsis suecica hits
. . Arabidopsis halleri	eudicots	410	1	Arabidopsis halleri hits
. . Capsella rubella	eudicots	394	4	Capsella rubella hits
. . Arabis nemorensis	eudicots	372	2	Arabis nemorensis hits
. Tarenaya hassleriana	eudicots	153	1	Tarenaya hassleriana hits

Figure S3. BLAST Result Summary.
